# Dental Societies

**Published:** 1877-06

**Authors:** K. B. Davis

**Affiliations:** Springfield, Ill.


### DENTAL SOCIETIES.

*Read before the Iowa State Dental Society.*

BY K. B. DAVIS, SPRINGFIELD, ILL.

MR. PRESIDENT AND GENTLEMEN:—We live in an age at once important, eventful, progressive, and utilitarian; one which constitutes a great epoch in the cycle in which time is now advancing. It casts its brightening glories before, and most significantly reveals to the ken of thinking man the exalted destiny which will be his. It is the soil in which is planted the millennial tree, whose roots are striking deep, and whose branches are rising and spreading to shelter mankind.

There are two kinds of progress especially prominent in our times.

These relate, first to the achievements of mind, and, secondly to liberal sentiment.

Mentality is rising. It is employed upon the noblest subjects which can engage the attention of man. Mind is coming in contact with mind, and new channels of thought are being struck out.

Facts are collected and generalized, principle upon principle educed, and truth accumulated from the contact of mind with mind, preceeds harmonious development and progress. Thought is falling in with thought, truth, with truth, and the stream has begun to move, and is gathering force, and volume, and purity, as it proceeds.

To what agencies shall we ascribe these liberal and progressive ideas that now so pre-eminently characterize the learned professions, and especially that of dentistry?

At no time in the history of the world has there been so much time, labor, and energy devoted to the formation and maintenance of societies for mutual aid and progressive development as at the present.

There are three leading elements that have been mainly instrumental in the unsurpassed progress and development of our profession.

They are *Dental Journals*, *Dental Colleges*, and *Dental Societies.* But of the two former we do not now propose to speak. And as much as we are indebted to our colleges and Journals for what they have done for the profession, all will, I think agree, that to the various societies scattered all over this land should the honor be given of exerting a more marked and direct influence in the enhancement of dental science.

In order that we may see more clearly what has been done in this direction, let us go back thirty years and see what was the condition of the profession at that time.

Prior to 1840, there was but little fellowship, public or private, between dental practitioners, and every acquisition was hoarded up for private use, and the secrets of the laboratory and operating room were never exhibited to a fellow practitioner for any consideration whatever.

Is it any wonder that, under this supreme state of illiberal bigotry, no material progress should have been made in all the years that preceeded the organization of the first American Dental Society in 1840?

This society exerted a great influence in dispelling that selfish system of exclusiveness, which denied to others the benefits of individual experiment, and in time, though by slow degrees, this exclusiveness became a reproach, and the result was a growth of sympathy of thought, of a community of desires and interests more in harmony with the spirit of the age, and the demands of a liberal humanity. From the organization of this society dates the dawning of that spirit of liberality which now pervades the profession, and which, more than all else, has made it what it is—useful, honorable, dignified, progressive, learned, and humane.

In 1844 another dental society was formed, called the "Mississippi Valley Dental Association," embracing the country west of the Allegheny ridge. In 1855 the "American Dental Convention" was organized; with open doors and outstretched arms it still welcomes any dentist who wishes to attend. From a desire for a higher tone in the investigation of the various departments of dental science, delegates from the local societies that then existed, formed the 'American Dental Association' at Niagara Falls in 1859, which was to be a delegate body, made up of the representative men of the profession. Since its organization, local societies have sprung up all over the country, and thus have augmented the membership of this society, as well as secured great improvement at home.

There are at the present time two national societies, which meet annually, twenty one State societies, that meet annually or semi-annually, and forty-five local and district societies, meeting monthly or semi-annually.

Who can estimate the mighty impulse of all these societies upon the entire profession, so far as its members place themselves under their fostering care and beneficent influence?

And this brings us to a consideration of the direct and immediate benefits to each member of the profession who is a habitual attendant of one or more dental societies. Whatever general effect is produced by these societies upon the profession at large, they of course, have the same effect upon each individual member of the profession, that avails himself of the privileges and benefits to be derived therefrom. The object of these societies is to increase professional knowledge, both theoretical and practical, to bring together all worthy operators so that they may know each other better, and thus be prepared to forbear one another, and to act in closer accord and harmony in the elevation and development of our profession. Hence, every operator is expected to come up and contribute from his stock of experience, observation, and knowledge, for the common good of all; thus securing a free interchange of sentiment, learning each others' difficulties, and assisting in their removal—creating a mutual sympathy and confidence, that is ever the offspring of personal acquaintance and friendship. By this interchange of sentiment new ideas are conceived, pondered, and brought forth, that have placed in the hands of many the key of success, for lack of which they had groped along in darkness and doubt in relation to many things, for which they can now thank some one of our dental societies.

They have sent many hearts back to their operating rooms full of the inspirations of hope. They have imparted to each one the collected wisdom of all; thus opening fountains of knowledge that are free to all who wish to partake of their delicious waters. They have so held up the true mirror of excellence in operating that all could see in what it consisted, thus enabling the public to judge and act more wisely in the selection of their dentist. Here popular errors in theory are corrected, and inferior methods of operating are amended, while new applications of old truths, methods, and principles in dental philosophy are vindicated.

But the daily clinics that are held by our societies have doubtless accomplished more, in a practical point of view, than any other exercise connected with them. They have lifted the mist and fog of doubt and perplexity from many whose dental pupilage and after experience and observation had not afforded them such opportunities as they had always desired to possess.

Our colleges are doing a great work in this direction, but they reach so few of the profession at large, that we must depend to a great extent, at least for the present, upon our dental societies for the practical benefits of clinical instruction.

Many do not really grasp the truth when brought out in discussion alone,—but couple the principle and method of application, and clinically demonstrate them to the eye, and they are at once fixed in the mind to be utilized in our offices in the daily routine of the future.

These clinics are conducted by some of our best and most experienced operators; thus giving to the young and less experienced operators the benefits of their accumulated wisdom and experience, in the preparation of cavities, the application of the rubber dam, and the insertion and finishing of difficult fillings. How many have, at these clinics, been inspired with a laudable ambition to excel in operating, by witnessing some of those golden gems, golden corners, golden halves, and even golden crowns, put in and condensed as solid as gold coin itself, and then compare these operations, the astonishment and wonder of the performer himself, with some of those musty and dingy stuffings which some of us may have seen, and which we hoped might stay in—at least till the fee was received, and the patient had gone.

Having briefly adverted to what has been done by our societies for the profession at large, and to all who have availed themselves of their benefits individually, it now remains for us to see, at least some of the things to be accomplished by them. To many, a great deal of the very best work done by our societies may seem fruitless of any good results; however, we are convinced that ultimately there will be a rich reward. Good, honest, and industrious efforts will at last be crowned with success. If we did not believe this, our enthusiasm for the dental profession might possibly fail us in some of the dark days that necessarily surround our pathway. If we had never had any darkness, we could not appreciate the value of the light.

In order that our societies may be more fruitful, more elevating and progressive with the entire profession as well as the individual members, we must induce every worthy operator in the profession to attend some one or more of our societies. Of the 14000 dentists in the United States, perhaps not more than 4000 at most, ever pretend to meet with any dental society. Perhaps some think they cannot afford to attend them. To such I would reply that did they know their true value, they would readily come to the conclusion that they could not afford to stay away. Others stay away from a general indifference, and the want of a proper knowledge of the societies. Still another class stay away because they are already so wise in the their own estimation that no society can teach them any thing more. What a happy cousummation! With what ineffable disdain must they look down from their proud and lofty position, to view us less favored mortals, who are constantly toiling onward and upward but still see that much desired state—perfection, far in the dis tance. To this class of dentists we would suggest that, if they have really reached perfection in our profession, that they are the very ones above all others who should attend our societies, and impart to their less favored brethren some of their pre-eminent skill and knowledge. How truly does this illustrate the language of the poet:—

> "A little learning is a dangerous thing,
> Drink deep, or touch not the pierian spring."

Did the above mentioned classes of our profession fully realize the deep responsibilities and obligations resting upon them in relation to their duties to the people, it would seem that this would be sufficient to at once induce them to become the happy recipients of the benefits to be derived from a regular attendance at some one or more of our dental societies. It would seem that pecuniary considerations alone, if no higher motives were taken into consideration, ought to be sufficient to induce an interest in our societies; for, undoubtedly, all are desirous of both professional and pecuniary success. Who among our entire profession have achieved the greatest success? Has it not been those who have been, and are now, our most earnest and zealous workers, not only in one but several of our dental societies? Take the men that work in our societies away from the profession, and what would be the consequences? Why, it would, in a very short time, sink to the level where it was thirty years ago—a mere itinerant, catch-penny, tinkering business. Amalgam would again assert its supremacy, and the unrelenting forceps would hold undisputed sway over those priceless gems that give such matchless beauty to the human face.

I do not pretend to say that dental societies can readily transform every poor and indifferent operator into a paragon of perfection; but I do assert that they will make of every honest seeker of truth and progress a better dentist in every sense of the word than he can be without them. Nor do I deny that there may be good operators that do not attend these societies, but I think I may safely say without fear of contradiction that if they are good operators without the aid of a society, they would be much better operators by placing themselves under their influence. If the professors in our Colleges, the Editors of Journals, and all of the most eminent members of our profession consider these societies so essential to them in this day of rapid progress, what shall we say of their crowning necessity to the wants of the great masses of the profession who have not, as yet, been brought to a position where they can see and fully appreciate the benefits to be derived from them.

